# Actually, what *is* a gain-of-function mutation?

**DOI:** 10.1093/genetics/iyag059

**Published:** 2026-04-08

**Authors:** Tobias Warnecke

**Affiliations:** Department of Biochemistry, University of Oxford, Oxford OX1 3QU, United Kingdom; Trinity College, Oxford OX1 3BH, United Kingdom

**Keywords:** gain-of-function, loss-of-function, mutation

## Abstract

For more than a century, scientists have worked to characterize, understand, and predict the consequences of mutations. For almost as long, scientists—always on the lookout for general principles—have categorized these mutations, hoping that putting them into labeled boxes might help reveal the molecular logic that governs mutational effects. Here, I will dive into one of these boxes, labeled “gain-of-function”, a term that will ring familiar to undergraduates, (clinical) geneticists, and virologists alike. I will emerge from the box with a profound sense of bewilderment and the conclusion that its contents appear to have very little in common. What *is* a gain-of-function mutation? What do we know (or can reasonably assume) about a mutation once it has attracted this label? Do gain-of-function mutations share anything in common in terms of their molecular features or the consequences they cause? I will argue that the answers to these three questions are “I don’t know,” “not much,” and “not really,” and that the term gain-of-function tells us rather little. Worse, it often misleads our intuition regarding what a given mutation is or does. I will suggest that this is because the gain-of-function label has historically been applied, with liberal abandon, across different levels of biological complexity, from the behavior of individual proteins, to protein complexes, to cells, to whole-organism physiology. I will discuss the implications (all bad…) of this heterogeneous labeling history for recent efforts to train machine learning algorithms to discriminate different types of mutations. Above all, I hope to highlight that the myriad ways in which mutational effects can percolate through biological systems often defy easy categorization and that, while classifying things is often useful, it is best not to forget that molecular biology is a glorious mess.

In 1932, American geneticist Hermann Muller tried to make sense of it all. For years, he and others had been irradiating fruit flies with X-rays and observing the consequences. As the high-energy radiation fractured the flies’ DNA and caused individual fragments to be lost or reattached to other chromosomes, the appearance of the flies changed. This included a wide variety of changes—in eye color, wing size, or the number of bristles on the fly's thorax—but Muller proposed that there was some deeper logic and that mutations could be grouped based on the nature of their impact (Muller 1932): “Hypomorphic” mutations, he argued, were those that work “*in the same direction (towards the same superficial end result) as the normal allelomorph, but not so strongly,*” exemplified by *apricot* and *eosin* mutants, whose eyes were noticeably less red than the wildtype. These mutant genes did not actively *suppress* the generation of red pigment—adding more copies of the mutant gene led to more intense color—they simply produced less pigment. At the end of the hypomorphic cul-de-sac lay “amorphic” mutations, where the phenotype in question (e.g. red pigment) stopped being produced altogether. Mutations that led to augmentation of a trait, on the other hand, such as an increased number of bristles, Muller labeled “hypermorphic.” Here, adding more copies of the gene exacerbated the mutant phenotype. Creating hypermorphs (typically by overexpression or mis-expression, e.g. in a different tissue) and hypomorphs (by gene deletion or gene product depletion) remains a vital part of the geneticist's toolkit and a powerful general stratagem to dissect genetic pathways.

On occasion, the effects observed by Muller and his fellow fly-pushers were difficult to rationalize as an increase or decrease of gene dosage. Rather, they appeared to change “*the nature of the gene at the original locus, giving an effect not produced… by the original normal gene.*” These “neomorphic” mutations appeared to cause a qualitative rather than quantitative change and adding more copies of the wildtype gene did not alleviate the mutant phenotype. One of Muller's examples is the *bar* mutation, which affects not only the number but also the distribution of the ommatidia that form the fly's facet eyes, giving rise to a diagnostic strip-eyed look.

## Legacy of the neomorph

Fast forward a little less than a hundred years, and Muller's terms are no longer in widespread use. Look up “neomorph” in your favorite search engine, and you are likely to learn rather more about the film Alien (where a neomorph is an extraterrestrial endoparasitoid, in case you were wondering) than the eye color of mutant flies. This is not because people have stopped categorizing mutations by their impact. Far from it. But Muller's monikers have mostly been replaced by two broader terms, introduced as early as 1963 by developmental Drosophilist E. B. Lewis (Lewis 1963): hypo- and amorphic mutations are nowadays more likely to be called partial and full loss-of-function (LOF) mutations, respectively, while hyper- and neomorphic changes are usually lumped together under the label gain-of-function (GOF). There is no universally agreed definition of LOF and GOF mutations, but Muller's initial classification scheme continues to reverberate through an age where our understanding of the molecular basis of mutational effects has gotten much more granular:

Gain-of-function mutations are said to “*enhance the activity of the mutated protein*” (Bayrak et al. 2021), “*enhance or recruit new protein functions*” (Ge et al. 2022), lead to “*constitutive activation, shift of substrate or binding specificity or aggregation*” (Gerasimavicius et al. 2022) or, most all-embracing, to a protein that “*does something different than the wildtype protein*” (Backwell and Marsh 2022). GOF mutations are seen to *add* something, in either quantitative (hypermorphic) or qualitative (neomorphic) terms. Popular textbooks, too, place single proteins at the center of their gain-of-function definition, focusing on the consequences for the product of the gene in which the mutation resides, for example defining GOF as “the acquisition of a novel property by the mutant protein, or the expression of a gene at the wrong time (heterochronic expression) and/or in the wrong place (ectopic expression)” (Nussbaum et al. 2016). [NB: The discussion below covers both mutations and variants, i.e. genetic changes that have occurred in the germline and segregate in a population. For simplicity—and without loss of accuracy regarding the molecular case I am about to make—I will refer to both types of genetic changes as mutations throughout.]

But, based on how the term has been used in practice, gain-of-function can manifest at a number of levels of biological complexity: as a mutant ion channel with increased conductivity or a neuron that fires at a higher rate. Or—harking back to Muller's day—at the level of organismal morphology. This is particularly obvious in the clinical literature, where the need for categorization only arises in response to an observed human pathology. In short, there is a great deal of latitude, liberally exploited by practicing scientists, in how the gain-of-function label has been applied over time. This stretches the utility of the label in important ways. Notably, where the gain-of-function label is applied to a higher level of biological complexity (e.g. morphology), it is easily taken to imply some gain-of-function mechanism at a lower level (e.g. a protein's activity). As I will illustrate below, this intuition is often false.

Ok, sometimes the stars align and gain-of-function is understood with intuitive simplicity. This is the case, for example, in an area of research that has come close to usurping the term gain-of-function: work on the infectious capacity of viruses. Here, we are frequently dealing with mutations that lead proteins to establish a new or alter an old interaction with a host receptor (Meyer et al. 2012) or some component of the host membrane or cell wall (Imai et al. 2012), which then enables invasion or transmission between hosts. There is a gain in capacity for both the individual protein and the virus as a whole. But, as Muller realized early on, the link between molecular, cellular, and organismal phenotype is not always this straightforward.

## Your gain-of-function is my loss-of-function

Take ion channels. Here, the functional impact of a mutation (gain vs loss) is usually assigned based on what happens to channel conductivity. Across the vast literature on ion channel proteins, mutations are described as gain-of-function if they lead to slower inactivation kinetics (Albuisson et al. 2013; Ma et al. 2018), a constitutively open channel (Kim et al. 2007; Pavel et al. 2016), an increased channel-open probability (Rauh et al. 2013; Teng et al. 2015, 2016), a lower excitability threshold (Dib-Hajj et al. 2005; Li et al. 2009; Swan et al. 2014), or insensitivity to ligands that would normally lead to closing of the channel (Kim et al. 2007; Subbotina et al. 2019)—in short, any proximate mechanism that results in an increased flux of ions through the channel. But look deeper—to what happens to individual proteins and their interactions—and your expectation of finding something being gained will often be disappointed.

Let us consider the L596I mutation in the human ion channel protein TRPV4. This mutation, which sees a leucine at position 596 in the protein replaced by an isoleucine, increases the probability of channel opening, resulting in an increased flux of cations through the channel. It does so, however, not by establishing some new connection but by loosening a molecular latch that normally favors a closed channel conformation (Teng et al. 2015). No new interaction is gained. Rather, an old one is lost. Similarly, varitint-waddler mice—deaf and struggling with their balance—carry an A419P mutation in TRPML3, another cation channel protein (Kim et al. 2007). The mutation disrupts communication with a pH-sensing domain, locking the channel into a constitutively open state (Kim et al. 2008). For these mutations, and a plethora of others (Lester and Karschin 2000), it is hard to argue that the individual proteins affected have gained anything. Instead, increased channel conductivity is brought about by loss of an interaction or appropriate feedback control at the protein level.

## Removing the off switch

Evading normal control mechanisms is a common theme for gain-of-function mutations beyond ion channels. Let's rattle through some examples:

- certain mutations (e.g. R274Q) in STAT1—a transcription factor that helps coordinate cellular responses to signals from the immune system—interfere with STAT1 dephosphorylation. As a consequence, STAT1 is retained in the nucleus rather than shuttled back to the cytoplasm, ultimately causing chronic mucocutaneous candidiasis (Liu et al. 2011).
- mutations in the common docking domain of the MAP kinase ERK2 (e.g. D321N) are no longer recognized by their cognate phosphatases, leading to persistent activation, which can drive cell proliferation and cancer (Brunner et al. 1994; Robles et al. 2025).
- mutations in oncogenic Ras proteins typically lead to loss of their GTPase activity. This leads to constitutive activation of Ras, since it is the GTP-bound form that engages with downstream targets (Hobbs et al. 2016).
- mutating the K296/E113 salt bridge in the dim-light photoreceptor rhodopsin mimics the activated state of the receptor because the chromophore that would normally bind there to inhibit signaling (11-*cis-*retinal), no longer can (Kim et al. 2004), giving rise to retinitis pigmentosa.
- certain cancer-associated mutations in the protein phosphatase 1D (PPM1D/WIP1) destroy a degradation signal (degron). This stabilizes the protein, which negatively regulates the tumor suppressor p53, thereby promoting tumor formation (Tokheim et al. 2021).

Bottom line: molecular behavior that, quite reasonably, has been described as gain-of-function frequently emerges from what a reasonable person (i.e. me) would describe as loss-of-function at a lower level of complexity or at an earlier stage of a molecular cascade, and is often manifest as a loss-of-responsiveness to its normal regulators.

## Gain to lose

Conversely, a card-carrying gain-of-function at the protein level, such as when a protein establishes a new protein-protein interaction, can underpin loss-of-function further down the track.

Consider [RNQ+], the prion form of yeast protein Rnq1. Overexpression of [RNQ+]—not a genetic mutant, granted, but please indulge me—leads to cell cycle arrest. But this is not because [RNQ+] deleteriously boosts a pathway in which it normally acts. Rather, the prion, present in greater number and eminently without anything better to do, begins to seek interactions outside its normal friendship circle. In particular, it starts to interact with Spc42, a key member of the spindle pole body, compromising its normal function (Treusch and Lindquist 2012). [RNQ+] is not the only protein that exerts a bad influence on new, impressionable friends. Emergent toxic relationships like this, where newly promiscuous protein A sequesters protein B, often outside its normal pathway, appear to be common, especially for proteins with the capacity to form amyloid structures (Olzscha et al. 2011) or those with intrinsically disordered regions (IDRs) (Vavouri et al. 2009; Mosca et al. 2012).

For example, polyQ-expanded versions of the huntingtin protein co-aggregate with proteins that also contain (smaller) glutamine-rich stretches but that would not aggregate and form amyloids by themselves. One of these proteins is CREB binding protein (CBP) and the pathological consequences of polyQ expansion are, at least in part, likely down to sequestration of CBP (Jiang et al. 2006; Holmes et al. 2014). Similarly, the gain of a dileucine motif in an IDR of the glucose transporter protein GLUT1 gives rise to a new interaction with clathrin. This gain-of-function then causes loss-of-function of the transporter, which, whisked away by clathrin, ends up in the wrong part of the cell (Meyer et al. 2018).

The simple take-home here is that whether we label a mutation as loss- or gain-of-function usually depends on where we choose to look. In the cases highlighted above, there is no denying that interactions at the protein level are gained or reinforced, but zooming out and looking at what happens downstream of this interaction, we can be equally assertive in stating that these mutations cause a loss-of-function, albeit not in the protein's normal stomping ground. The loss is happening off-pathway (which, incidentally, makes the physiological consequences of this type of mutation much harder to predict).

## United in opposition

Given the diversity of mechanisms and levels of biological complexity to which the term gain-of-function has been applied, is there anything that unites gain-of-function mutations? Are there any general mechanistic lessons we can learn that go beyond the idiosyncrasies of individual mutations?

Multiple recent studies have attempted to address this very question, using a variety of machine learning approaches to identify features that discriminate gain-of-function from loss-of-function mutations, and from mutations that are benign (in the sense that they are present in a population of ostensibly healthy humans) (Lee et al. 2009; Jung et al. 2015; Heyne et al. 2020; Bayrak et al. 2021; Gerasimavicius et al. 2022; Stein et al. 2023; Badonyi and Marsh 2024, 2025). The starting point for these endeavors are curated sets of mutations, which have been labeled as gain- or loss-of-function in clinical databases (e.g. OMIM, ClinVar) in all the spectacularly heterogeneous, inconsistent, and level-of-complexity-agnostic ways that we have encountered above. So, there should be little hope for finding *any* unifying features of gain-of-function mutations. Right?

Wrong. The various algorithms implemented in these studies do an admirable job of discriminating gain- from loss-of-function mutations and have highlighted a number of residue-, gene-, and protein-level features, alongside dominant/recessive inheritance patterns (Box 1), that power accurate discrimination. Most notably, mutations that strongly destabilize a protein or eliminate protein expression altogether (e.g. via premature termination codons that trigger nonsense-mediated decay) are more likely to be associated with the loss-of-function label (Jung et al. 2015; Gerasimavicius et al. 2020, 2022; Bayrak et al. 2021; Stein et al. 2023). In contrast, changes associated with gain-of-function mutations are milder; the mutant protein causes problems not by disappearing, but by sticking around.

Box 1. **Gain-of-function versus dominance**

Loss-of-function and gain-of-function labels are associated, and sometimes conflated, with two other labels: recessive and dominant, respectively (Bayrak et al. 2021; Gerasimavicius et al. 2022). In contrast to the vague definitions of GOF and LOF (see main text), the definition of dominance is much tighter: it is the property of a trait or character, where the character is said to be dominant when seen in a heterozygote and recessive if only observed in a homozygous state, with predictive implications for how traits are inherited. Dominance is therefore not a property of a single gene or protein or mutation, but a property of the genetic system describing how two alleles interact in their effect on a focal phenotype. By definition, dominance is intimately tied to the biology of diploid (or polyploid) biological systems. In contrast, GOF/LOF labels can readily be applied to monoploid systems, which include a vast swath of prokaryotes.

While conceptually distinct, the two sets of descriptors (GOF/LOF vs dominant/recessive) are nonetheless related, in that one is often predictive of the other (Bayrak et al. 2021; Gerasimavicius et al. 2022). Empirically, loss-of-function mutations are often recessive; taking out one copy is not enough to compromise the phenotype. Why this so-called haplosufficiency would be a common property of diploid molecular systems is a curious and nontrivial question that has occupied scientific minds far greater than mine, notably including R.A. Fisher and Sewall Wright. Conversely, gain-of-function mutations are typically dominant—and perhaps sometimes labelled as GOF solely thanks to their dominant inheritance.

There are plenty of exceptions to the LOF = recessive/GOF = dominant mapping, most notably LOF mutations in haploinsufficient genes and dominant negative mutations, the classic example for which are mutations in proteins that form homo-oligomeric complexes, where one nonfunctional allele can poison the entire complex and thereby compromise the function of >50% of proteins. Dominant negative mutations, for which Muller had reserved the special term *antimorphic* (Muller 1932), perfectly encapsulate the schizophrenic nature of the labelling scheme: being both gain of function (at the protein level) and loss of function (at the phenotypic level) at the same time.

Conversely, card-carrying GOF mutations can be recessive. For example, some mutations (e.g. T4706M) in the ryanodine receptor 1 (RYR1) gene act by generating a constitutively high channel opening probability (Brennan et al. 2019; Magyar et al. 2023). Following the precedence set by other ion channels, these mutations, which are associated with malignant hyperthermia, squarely deserve the GOF label. However, these mutations often show a recessive inheritance pattern as a single mutated allele is insufficient to cause a clinically observable phenotype. Another example is Chuvash polycythemia, where the R200W mutation in the VHL (von Hippel-Landau) protein results in overactive HIF1 signaling (Ang et al. 2002). Again, a phenotype is only observable when both copies of the VHL gene are mutated, resulting in a GOF mutation with a recessive inheritance pattern. This example illustrates nicely the broader issue here: the R200W mutation has been labelled as a GOF mutation based on the increase in HIF1 signaling. At a lower level of complexity, however, a LOF label would be equally appropriate as the R200W mutations abolish the interaction between VHL and HIF1.

As common features go, however, “being around” is a pretty low bar to clear. Mutations within this set remain hugely diverse mechanistically. I would argue that in a direct head-to-head against loss-of-function mutations, gain-of-function mutations are defined not by what they are but by what they are not. They are not (for the most part) mutations that lead to loss of the protein or where normality can be restored through complementation (i.e. by supplying a wildtype copy of the gene). They are united only in opposition, as non-LOFs (Gerasimavicius et al. 2022). It is unsurprising, then, that differentiating gain-of-function mutations (as labeled in the literature) from benign missense substitutions—the true acid test of whether gain-of-function mutations share anything in common that makes them stand out from the crowd—has proven noticeably harder (Bayrak et al. 2021; Stein et al. 2023). And even where apparent predictive power remains, I wonder whether biases in the training data are what drives classification success, e.g. gain-of-function mutations in some ion channels predicting gain-of-function mutations in some other ion channels (Jung et al. 2015; Bayrak et al. 2021; Gerasimavicius et al. 2022; Badonyi and Marsh 2025).

## Evasive fireflies in the archaic night

Assigning labels is always a balancing act. As humans, we are intimately familiar with both the convenience (tomatoes are in the vegetable isle, making shopping for all your salad needs more efficient) and dangers (people making fun of you because you don’t know that “tomatoes really are a fruit”… whatever, man.) labeling provides. Despite the pitfalls, categorizing, labeling, and trying to impose order on the observable universe have been central to the scientific pursuit well before Linnaeus really took the handbrake off. As Camille Paglia put it:

“Western science is a product of the Apollonian mind: its hope is that by naming and classification, by the cold light of intellect, archaic night can be pushed back and defeated.” (Paglia 1991).

At their best, labels allow us to see beyond the individual instance, to discover broader patterns and the underlying, hidden processes that govern them. In the modern world, labels have been essential to train classifiers that allow automated and highly accurate discrimination of cats from dogs (https://www.kaggle.com/competitions/dogs-vs-cats/overview), and dogs from muffins (https://www.kaggle.com/datasets/samuelcortinhas/muffin-vs-chihuahua-image-classification).

At their worst, however, labels might lump together things that are different in many important ways, blinding us to the diversity within. This is certainly true for algorithmic classifiers where limited options are enforced from the outset. If you are trained to discriminate dogs from cats, there is only so much we can expect when handing you the picture of a penguin (although, according to my learned colleague Dr Hocher, penguins are “clearly closer to cats”).

The tension between seeking to establish order and putting two very different things into the same bin simply because we have a self-imposed limit on the number of bins very much applies to classifying mutations. Gain-of-function mutations, in this bin-based analogy, go in the general waste container, marked out only by the fact that they do not readily conform to a narrower category like recyclable cardboard.

This is not to say that the gain-of-function label has been unreasonably applied in any one particular instance. But its past and current application comprises such a diversity of mechanisms and levels of biological complexity that I find it challenging to think about gain-of-function mutations as a class unified by particular features across proteins and contexts and I worry that the label might unwittingly make us lose sight of the complexity of the underlying biology or make us short-cut to the assumption that gain at the level of, say, ion channel conductivity, implies gain of a molecular interaction at the protein level.

Where do all these musings leave us? For me, the label gain-of-function lives on the edge of utility. Inconsistent past and present application to different levels of biological complexity make it a treacherous rather than intuitive guide to function. There is value in discriminating gain- and loss-of-function in specific contexts, where transfer learning might provide generalizable mechanistic insights (Heyne et al. 2020; Zhong et al. 2025). But if we want to deploy machine learning approaches more broadly to predict the nature of mutational effects within a GOF/LOF framework, we will need conceptually precise and consistent labels. To accomplish this, and to move beyond the constraints of historically inconsistent labeling, we will probably need to tip out the general waste container and try to have another go at sorting its contents.

With my Utopian hat on, I might suggest that there is value in embarking on a systematic re-labeling effort, where GOF/LOF labels are applied to a defined level of biological complexity, the single protein level being perhaps the obvious one. Or, and without appearing nostalgic of a past I never experienced, I might advocate for a return to a more strictly Mullerian definition, where GOF and LOF labels are anchored to dosage effects, and complementation via a wildtype copy would be an acid test for what constitutes a loss-of-function mutation. But my Utopian hat is a couple of sizes too large and tends to slip over my eyes, blinding me to reality. So I don’t wear it all that often. Without it, any aspiration for a systematic re-labeling effort starts to look rather delusional. Aside from strong field-specific conventions in how and at what level of complexity GOF/LOF labels are applied (remember ion channels), the labels in their current incoherent incarnations are deeply embedded and will continue to be applied in the clinical literature, where well-controlled functional studies and complementation assays are—while desirable—often impossible.

Speaking of “functional studies,” the term “function” in and of itself is, of course, tricky. We can spend hours advocating for a causal role definition (function is what the protein does) or an evolutionary definition (where selection for the presence of a trait is seen as the ultimate arbiter of what is functional), in a discussion that will likely be as animated as it will be unproductive (Keeling et al. 2019). I’d rather not.

For now, I simply live in the vain hope that the odd biology student will stumble upon this essay to find a counterpoint to ChatGPT-generated summaries, issued with seductive authority, of what a gain-of-function mutation is and to find yet another example of the fact that—cliché alert—biology is complex.

## References

[iyag059-B1] Albuisson J et al 2013. Dehydrated hereditary stomatocytosis linked to gain-of-function mutations in mechanically activated PIEZO1 ion channels. Nat Commun. 4:1884. 10.1038/ncomms2899. 23695678 PMC3674779

[iyag059-B2] Ang SO et al 2002. Disruption of oxygen homeostasis underlies congenital chuvash polycythemia. Nat Genet. 32:614–621. 10.1038/ng1019. 12415268

[iyag059-B3] Backwell L, Marsh JA. 2022. Diverse molecular mechanisms underlying pathogenic protein mutations: beyond the loss-of-function paradigm. Annu Rev Genomics Hum Genet. 23:475–498. 10.1146/annurev-genom-111221-103208. 35395171

[iyag059-B4] Badonyi M, Marsh JA. 2024. Proteome-scale prediction of molecular mechanisms underlying dominant genetic diseases. PLoS One. 19:e0307312. 10.1371/journal.pone.0307312. 39172982 PMC11341024

[iyag059-B5] Badonyi M, Marsh JA. 2025. Prevalence of loss-of-function, gain-of-function and dominant-negative mechanisms across genetic disease phenotypes. Nat Commun. 16:8392. 10.1038/s41467-025-63234-3. 40998763 PMC12462468

[iyag059-B6] Bayrak CS et al 2021. Identification of discriminative gene-level and protein-level features associated with pathogenic gain-of-function and loss-of-function variants. Am J Hum Genet. 108:2301–2318. 10.1016/j.ajhg.2021.10.007. 34762822 PMC8715146

[iyag059-B7] Brennan S et al 2019. Mouse model of severe recessive RYR1-related myopathy. Hum Mol Genet. 28:3024–3036. 10.1093/hmg/ddz105. 31107960 PMC6737254

[iyag059-B8] Brunner D et al 1994. A gain-of-function mutation in Drosophila MAP kinase activates multiple receptor tyrosine kinase signaling pathways. Cell. 76:875–888. 10.1016/0092-8674(94)90362-x. 8124723

[iyag059-B9] Dib-Hajj SD et al 2005. Gain-of-function mutation in Nav1.7 in familial erythromelalgia induces bursting of sensory neurons. Brain. 128:1847–1854. 10.1093/brain/awh514. 15958509

[iyag059-B10] Ge F et al 2022. VPatho: a deep learning-based two-stage approach for accurate prediction of gain-of-function and loss-of-function variants. Brief Bioinform. 24:bbac535. 10.1093/bib/bbac535. 36528806

[iyag059-B11] Gerasimavicius L, Liu X, Marsh JA. 2020. Identification of pathogenic missense mutations using protein stability predictors. Sci Rep. 10:15387. 10.1038/s41598-020-72404-w. 32958805 PMC7506547

[iyag059-B12] Gerasimavicius L, Livesey BJ, Marsh JA. 2022. Loss-of-function, gain-of-function and dominant-negative mutations have profoundly different effects on protein structure. Nat Commun. 13:3895. 10.1038/s41467-022-31686-6. 35794153 PMC9259657

[iyag059-B13] Heyne HO et al 2020. Predicting functional effects of missense variants in voltage-gated sodium and calcium channels. Sci Transl Med. 12:556. 10.1126/scitranslmed.aay6848. 32801145

[iyag059-B14] Hobbs GA, Der CJ, Rossman KL. 2016. RAS isoforms and mutations in cancer at a glance. J Cell Sci. 129:1287–1292. 10.1242/jcs.182873. 26985062 PMC4869631

[iyag059-B15] Holmes WM, Klaips CL, Serio TR. 2014. Defining the limits: protein aggregation and toxicity in vivo. Crit Rev Biochem Mol Biol. 49:294–303. 10.3109/10409238.2014.914151. 24766537 PMC4238936

[iyag059-B16] Imai M et al 2012. Experimental adaptation of an influenza H5 HA confers respiratory droplet transmission to a reassortant H5 HA/H1N1 virus in ferrets. Nature. 486:420–428. 10.1038/nature10831. 22722205 PMC3388103

[iyag059-B17] Jiang H et al 2006. Depletion of CBP is directly linked with cellular toxicity caused by mutant huntingtin. Neurobiol Dis. 23:543–551. 10.1016/j.nbd.2006.04.011. 16766198

[iyag059-B18] Jung S, Lee S, Kim S, Nam H. 2015. Identification of genomic features in the classification of loss- and gain-of-function mutation. BMC Méd Inform Decis Mak. 15:S6. 10.1186/1472-6947-15-s1-s6. PMC4460711 26043747

[iyag059-B19] Keeling DM, Garza P, Nartey CM, Carvunis A-R. 2019. The meanings of “function” in biology and the problematic case of de novo gene emergence. eLife. 8:e47014. 10.7554/elife.47014. 31674305 PMC6824840

[iyag059-B20] Kim J-M et al 2004. Structural origins of constitutive activation in rhodopsin: role of the K296/E113 salt bridge. Proc Natl Acad Sci U S A. 101:12508–12513. 10.1073/pnas.0404519101. 15306683 PMC515088

[iyag059-B21] Kim HJ et al 2007. Gain-of-function mutation in TRPML3 causes the mouse varitint-waddler phenotype*. J Biol Chem. 282:36138–36142. 10.1074/jbc.c700190200. 17962195

[iyag059-B22] Kim HJ et al 2008. A novel mode of TRPML3 regulation by extracytosolic pH absent in the varitint-waddler phenotype. EMBO J. 27:1197–1205. 10.1038/emboj.2008.56. 18369318 PMC2367400

[iyag059-B23] Lee W et al 2009. Bi-directional SIFT predicts a subset of activating mutations. PLoS One. 4:e8311. 10.1371/journal.pone.0008311. 20011534 PMC2788704

[iyag059-B24] Lester HA, Karschin A. 2000. Gain of function mutants: ion channels and G protein-coupled receptors. Annu Rev Neurosci. 23:89–125. 10.1146/annurev.neuro.23.1.89. 10845060

[iyag059-B25] Lewis EB . 1963. Genes and developmental pathways. Am Zoöl. 3:33–56. 10.1093/icb/3.1.33.

[iyag059-B26] Li Q et al 2009. Gain-of-function mutation of Nav1.5 in atrial fibrillation enhances cellular excitability and lowers the threshold for action potential firing. Biochem Biophys Res Commun. 380:132–137. 10.1016/j.bbrc.2009.01.052. 19167345

[iyag059-B27] Liu L et al 2011. Gain-of-function human STAT1 mutations impair IL-17 immunity and underlie chronic mucocutaneous candidiasis. J Exp Med. 208:1635–1648. 10.1084/jem.20110958. 21727188 PMC3149226

[iyag059-B28] Ma S et al 2018. Common PIEZO1 allele in African populations causes RBC dehydration and attenuates plasmodium infection. Cell. 173:443–455.e12. 10.1016/j.cell.2018.02.047. 29576450 PMC5889333

[iyag059-B29] Magyar ZÉ et al 2023. Function of a mutant ryanodine receptor (T4709 M) linked to congenital myopathy. Sci Rep. 13:14659. 10.1038/s41598-023-41801-2. 37670077 PMC10480487

[iyag059-B30] Meyer JR et al 2012. Repeatability and contingency in the evolution of a key innovation in phage lambda. Science. 335:428–432. 10.1126/science.1214449. 22282803 PMC3306806

[iyag059-B31] Meyer K et al 2018. Mutations in disordered regions can cause disease by creating dileucine motifs. Cell. 175:239–253.e17. 10.1016/j.cell.2018.08.019. 30197081

[iyag059-B32] Mosca R, Pache RA, Aloy P. 2012. The role of structural disorder in the rewiring of protein interactions through evolution*. Mol Cell Proteom. 11:M111.014969-1–M111.014969-8. 10.1074/mcp.m111.014969. PMC3394948 22389433

[iyag059-B33] Muller HJ . 1932. Further studies on the nature and causes of gene mutations. In: Proceedings of the 6th International Congress of Genetics; Ithaca, New York. Brooklyn Botanic Garden, Brooklyn, NY; Composite re-print of complete proceedings: Genetics Society of America, Austin, TX. p 213–255.

[iyag059-B34] Nussbaum RL, McInnes RR, Willard HF. 2016. Thompson & Thompson Genetics in Medicine. 8th Ed Elsevier/Saunders.

[iyag059-B35] Olzscha H et al 2011. Amyloid-like aggregates sequester numerous metastable proteins with essential cellular functions. Cell. 144:67–78. 10.1016/j.cell.2010.11.050. 10.1016/j.cell.2010.11.050. 21215370

[iyag059-B36] Paglia C . 1991. Sexual personae: art and decadence from Nefertiti to Emily Dickinson. Random House/Vintage Books.

[iyag059-B37] Pavel MA et al 2016. Function and regulation of TRPP2 ion channel revealed by a gain-of-function mutant. Proc Natl Acad Sci U S A. 113:E2363–E2372. 10.1073/pnas.1517066113. 27071085 PMC4855601

[iyag059-B38] Rauh R et al 2013. A mutation in the β-subunit of ENaC identified in a patient with cystic fibrosis-like symptoms has a gain-of-function effect. Am J Physiol Lung Cell Mol Physiol. 304:L43–L55. 10.1152/ajplung.00093.2012. 23087020

[iyag059-B39] Robles JT, Stiegler AL, Boggon TJ, Turk BE. 2025. Cancer hotspot mutations rewire ERK2 specificity by selective exclusion of docking interactions. J Biol Chem. 301:108348. 10.1016/j.jbc.2025.108348. 40015635 PMC11982978

[iyag059-B40] Stein D et al 2023. Genome-wide prediction of pathogenic gain- and loss-of-function variants from ensemble learning of a diverse feature set. Genome Med. 15:103. 10.1186/s13073-023-01261-9. 38037155 PMC10688473

[iyag059-B41] Subbotina E et al 2019. Functional characterization of ABCC9 variants identified in sudden unexpected natural death. Forensic Sci Int. 298:80–87. 10.1016/j.forsciint.2019.02.035. 30878466 PMC6527451

[iyag059-B42] Swan H et al 2014. Gain-of-function mutation of the SCN5A gene causes exercise-induced polymorphic ventricular arrhythmias. Circ Cardiovasc Genet. 7:771–781. 10.1161/circgenetics.114.000703. 25210054

[iyag059-B43] Teng J, Loukin SH, Anishkin A, Kung C. 2015. L596–W733 bond between the start of the S4–S5 linker and the TRP box stabilizes the closed state of TRPV4 channel. Proc Natl Acad Sci U S A. 112:3386–3391. 10.1073/pnas.1502366112. 25737550 PMC4371962

[iyag059-B44] Teng J, Loukin SH, Anishkin A, Kung C. 2016. A competing hydrophobic tug on L596 to the membrane core unlatches S4–S5 linker elbow from TRP helix and allows TRPV4 channel to open. Proc Natl Acad Sci U S A. 113:11847–11852. 10.1073/pnas.1613523113. 27698146 PMC5081603

[iyag059-B45] Tokheim C et al 2021. Systematic characterization of mutations altering protein degradation in human cancers. Mol Cell. 81:1292–1308.e11. 10.1016/j.molcel.2021.01.020. 33567269 PMC9245451

[iyag059-B46] Treusch S, Lindquist S. 2012. An intrinsically disordered yeast prion arrests the cell cycle by sequestering a spindle pole body component. J Cell Biol. 197:369–379. 10.1083/jcb.201108146. 22529103 PMC3341155

[iyag059-B47] Vavouri T, Semple JI, Garcia-Verdugo R, Lehner B. 2009. Intrinsic protein disorder and interaction promiscuity are widely associated with dosage sensitivity. Cell. 138:198–208. 10.1016/j.cell.2009.04.029. 10.1016/j.cell.2009.04.029. 19596244

[iyag059-B48] Zhong G et al 2025. PreMode predicts mode-of-action of missense variants by deep graph representation learning of protein sequence and structural context. Nat Commun. 16:7189. 10.1038/s41467-025-62318-4. 40764308 PMC12325985

